# Supplementary material for: Observable Symptoms of Anxiety in Individuals with Fragile X Syndrome: Parent and Caregiver Perspectives
Source: Genes (Basel). 2022 Sep 16;13(9):1660. doi: 10.3390/genes13091660 (PMC9498703; doi:10.3390/genes13091660)
Supplement: Supplementary file 1 [file genes-13-01660-s001.zip › genes-1911730-supplementary file S1.pdf]

Forced Choice: List of Behavioral/Verbal Symptoms and Physical Symptoms

Behavioral/Verbal Symptoms

Aggression – Yells or hits other people or things, destroys property
Avoidance – Runs away, hides, refuses to go places, or come out of room, stays off to the side when in a group, becomes quiet, does not talk
Facial change - Appears angry, worried, or fearful
Fidgets – Makes small movements with hands, plays with a toy/item, and turns it over and over
Freezing – Unable to perform or give an answer
Hyperactive/Increased physical activity – Cannot sit still, goes for a walk
Nervous activities – Chewing gum, eating food/snack, drinking water/tea
Not wanting to do task/refusing to do activity
Pacing - Walks back and forth
Repetitive movements – Slams doors, repeats an action over and over
Resistance – Refuses to do things, gets on floor, and will not move or do anything
Self-injurious behaviors – Bites/hits/scratches self; bangs head
Speech becomes negative – Rapid, loud, growling, yelling, use of bad language, makes demands
Speech becomes repetitive - Says the same thing over and over, asks the same question repeatedly
Throws items

Physical Symptoms

Diarrhea/multiple bowel movements a day
Flushing/flushed face/red ears
Hiccups
Hives
Increased/rapid heart rate
Obvious muscle or body tension
Rapid breathing
Shakiness
Stomachache
Sweating
Vomiting
Zoning out

Wording of Survey

Describing Anxiety for People with Fragile X Syndrome

Welcome

Anxiety has been identified as one of the biggest issues most commonly interfering with functioning in individuals and families with Fragile X syndrome (FXS) – and this is based on the physical, behavioral, and verbal symptoms that parents see and hear.

Both families and clinicians rate anxiety as the most common limiting issue for those with FXS.

Most individuals with FXS cannot tell others they have anxiety, nor can they rate on a scale how much anxiety they have. Families and caregivers watch the individual's behavior and verbal output and describe that it seems the person with FXS is anxious.

Because anxiety is, by definition, an internal experience that an individual has to report him- or herself, the FDA (Food and Drug Administration - which approves clinical trials and new medications) has decided that caregiver-reported anxiety for a person with FXS cannot be used to prove if a medicine is working. YET, medications that reduce anxiety are needed to improve the quality of life of individuals with FXS.

So, we need families and caregivers to carefully describe how they know when someone with FXS is anxious (what they and others OBSERVE the person doing or saying to indicate their anxiety) and how it impacts their quality of life.

If families are able to do this, we can begin to develop scales or instruments that measure these behaviors. Eventually, we can use them to see if medicines are working for FXS.

In the survey below, we do ask you to differentiate between behavioral or verbal symptoms and physical symptoms. We have examples to help you. We also ask you to rate the frequency, intensity, and duration.

The survey is about people with the full mutation of FXS – including people who are mosaic with the full mutation.

Who can complete the survey:

Caregivers of individuals with FXS, including those who are mosaic.

Medical providers and other professionals who treat individuals with FXS.

Individuals with FXS who are able to self-determine their anxiety and complete the survey.

All ages and locations are eligible.

The survey can be completed once for each individual with FXS. So, if you have three children with FXS, you can complete it three times.

Thank you for participating in our survey!

Hint: If, at some point, you are unable to advance the survey, it automatically takes you to the point where information is missing.

Q1. Person completing this survey:

- Caregiver of someone with FXS.
- I have FXS.
- Professional who works with someone with FXS regularly or frequently.

Q2. Sex of person with FXS

- Male
- Female

Q3. Age, in years, of person with FXS – open.

Q4. For the purposes of this survey, when the words - anxiety or anxious - are used, it also includes when you think the person with FXS is worried or nervous or afraid.
In the person with FXS, would you say that at times the person is (check all that apply):

- Anxious/has anxiety
- Worried
- Nervous
- Afraid

Q5. Can the person with FXS tell you with their words that he/she is anxious?
(Does he/she actually say the words - for example, I am anxious about ____, I am worried about ____, I am nervous about ____, I am afraid about ____.)
If you are a professional who works with a person with FXS, is the person with FXS able to tell you with their words that he/she is anxious?

- No.
- Yes. If yes, what words does he/she use:

Q6. Please read this carefully.

Regardless of whether the person with FXS can state if he/she is anxious, we would like more detail about what you see and/or what you hear when you believe the person with FXS is anxious.

We want you to differentiate between these two areas: behavioral or verbal symptoms and physical symptoms.
We have separate questions below for each of the two areas.

Questions #6 - #10 are about the behavioral or verbal symptoms you observe when you believe the person with FXS is anxious.

_
When you believe the person with FXS is anxious-
What does he/she do? What does he/she **say?**
_

NOTE:
We are looking for the behavior or verbal symptoms when you believe the person with FXS is anxious – not behaviors related to other emotions like anger.

For each behavior or verbal sign listed below that you check "yes", please include the following

information in the drop-down boxes:

Frequency –
How often do you see this sign when the person with FXS is anxious?

Intensity –
How intense is this sign?

Duration –
How long does the sign last?

Reminder that this includes when the person with FXS is anxious, worried, nervous or afraid.

You only have to click - Yes - if it applies. You do not have to click on - No - if it does not apply.

If you select - Yes - you will have to complete - frequency, intensity, duration - for that sign, via the drop-down menus, in order to be able to move to the next page.

List of items:

Aggression – Yells or hits other people or things, destroys property

Avoidance – Runs away, hides, refuses to go places, or come out of room, stays off to the side when in a group, becomes quiet, does not talk

Facial change - Appears angry, worried, or fearful

Fidgets – Makes small movements with hands, plays with a toy/item, and turns it over and over

Freezing – Unable to perform or give an answer

Hyperactive/Increased physical activity – Cannot sit still, goes for a walk

Nervous activities – Chewing gum, eating food/snack, drinking water/tea

Not wanting to do task/refusing to do activity

Pacing - Walks back and forth

Repetitive movements – Slams doors, repeats an action over and over

Resistance – Refuses to do things, gets on floor, and will not move or do anything

Self-injurious behaviors – Bites/hits/scratches self; bangs head

Speech becomes negative – Rapid, loud, growling, yelling, use of bad language, makes demands

Speech becomes repetitive - Says the same thing over and over, asks the same question repeatedly

Throws items

Questions for each of the above items:

Do you see or hear this?

- Yes
- No

Frequency

- Rarely when they are anxious
- Sometimes when they are anxious
- About half the time when they are anxious
- Usually when they are anxious
- Always when they are anxious

Intensity

- Mild
- Slightly
- Moderately
- Very much
- Extremely

Duration

- Less than 1 minute
- 1 – 5 minutes
- 6 – 15 minutes
- 16 – 60 minutes
- Greater than 60 minutes

Q7. For this question, please list additional behavioral or verbal symptoms - not listed above - that you see or hear for this individual with FXS when you believe he/she is anxious.
List each additional sign on a separate line.
On question #8, you will be able to complete the frequency, intensity & duration for each additional behavioral or verbal sign you add here.

Q8. Describe the frequency, intensity & duration for the behavioral or verbal symptoms you added in Question #7.

Q9. On an average day, how many times do you see/hear the items in #6 & #7 above? Add them all together.

- 1-5 per day
- 6-10 per day
- 11-15 per day
- 16-20 per day
- >20 per day
- Comments

Q10. On a day when more or different activities/events are happening in the person's life, how many times a day do you see/hear the items in #6 & #7? Add them all together. Also mark if you see changes in intensity or duration.
Examples: Around the holidays, getting ready to go on vacation, when major transitions occur.

- 1-5 per day
- 6-10 per day
- 11-15 per day
- 16-20 per day
- >20 per day
- Increase in intensity
- Decrease in intensity
- Same intensity
- Increase in duration
- Decrease in duration
- Same duration
- Comments

Q11. Questions #11 – #15 are about the physical symptoms you observe when you believe the person with FXS is anxious. When you think the person with FXS is anxious – What physical symptoms do you see?

NOTE:
We are looking for the physical symptoms when you believe the person with FXS is anxious – not symptoms related to other emotions like anger.

For each physical sign listed below that you check "yes", please include the following information in the drop-down boxes:
Frequency – How often do you see this sign when the person with FXS is anxious?
Intensity – How intense is this sign?
Duration – How long does the sign last?
Reminder that this includes when the person with FXS is anxious, worried, nervous, or afraid.
You only have to click - Yes - if it applies. You do not have to click on - No - if it does not apply.
If you select - Yes - you will have to complete - frequency, intensity, duration - for that sign, via the drop-down menus, in order to be able to move to the next page.

List of items:

Diarrhea/multiple bowel movements a day
Flushing/flushed face/red ears
Hiccups
Hives

Increased/rapid heart rate

Obvious muscle or body tension

Rapid breathing

Shakiness

Stomachache

Sweating

Vomiting

Zoning out

Questions for each of the above items in Q11.

Do you see or hear this?

- Yes
- No

Frequency

- Rarely when they are anxious
- Sometimes when they are anxious
- About half the time when they are anxious
- Usually when they are anxious
- Always when they are anxious

Intensity

- Mild
- Slightly
- Moderately
- Very much
- Extremely

Duration

- Less than 1 minute
- 1 – 5 minutes
- 6 – 15 minutes
- 16 – 60 minutes
- Greater than 60 minutes

Q12. For this question, please list additional physical symptoms - not listed above - that you observe for this individual with FXS when you believe he/she is anxious. List each additional sign on a separate line.

On question #13, you will complete the frequency, intensity & duration for each additional physical sign you add here.

Q13. Describe the frequency, intensity & duration for the behavioral or verbal symptoms you added in Question #12.

14. On an average day, how many times do you see/hear the items in #11 & #12 above? Add them all together.

- 1-5 per day
- 6-10 per day
- 11-15 per day
- 16-20 per day
- >20 per day
- Comments

Q15. On a day when more or different activities/events are happening in the person's life, how many times a day do you see/hear the items in #11 & #12? Add them all together. Also mark if you see changes in intensity or duration.

Examples: Around the holidays, getting ready to go on vacation, when major transitions occur.

- 1-5 per day
- 6-10 per day
- 11-15 per day
- 16-20 per day
- >20 per day
- Increase in intensity
- Decrease in intensity
- Same intensity
- Increase in duration
- Decrease in duration
- Same duration
- Comments

Q16. The remaining questions ask about general information regarding the anxiety in the person with FXS.

With regards to the person with FXS - What is it about what you see or hear that you noted above that makes you confident he/she is anxious or has anxiety?

To put it another way - What specifically about the situation or the behavior itself makes you confident it is anxiety related?

Please elaborate.

Q17. When do you see the anxiety? Check all that apply.

- He/she appears anxious most of the day. Nothing specific, everything in general.
- Around specific scheduled daily events – school, work, day program.
- When he/she has to change from one activity to another (make a transition).
- When he/she knows a change/transition is coming up.

- When something new is going to happen, like an event that doesn't happen often like vacation.
- When something new is actually occurring, like when you are on vacation.
- When there is something that he/she doesn't want to do.
- When there is something unpleasant or that he/she doesn't want to happen or do in the future.
- When there are too many things happening all at once.
- When he/she is not sure what is going to happen next or how long it is going to last.
- When there are too many people around or it is too noisy or too bright.
- When he/she is going to a place where there might be too many people around or it may be too noisy or too bright.
- When there is no routine or schedule for the day.
- When there is an unresolved issue – a question that he/she has is left unresolved.
- Social situations or social demands, including meeting or facing new people.
- Other.

Q18. What helps to alleviate the anxiety? Check all that apply.

- Visuals/social stories – something visual that shows the person with FXS what is going to happen at an upcoming event.
- Talking through whatever the anxiety is about and/or helping to resolve issues.
- Allowing the person with FXS to observe the activity before joining/taking more time.
- Gross motor activities – Moving around – walking, running, swinging, jumping on a trampoline.
- Calming activities - listening to music, swinging, laying down, meditation.
- Medications daily use – like an SSRI or CBD.
- Medications single use – like Xanax or a homeopathic calming pill.
- Not doing the activity/event.
- Other.

Q19. How much do the behavioral/verbal and physical symptoms you identified above as showing anxiety impact the daily life of the person with FXS?

- Anxiety does not really limit functioning in any way.
- Anxiety limits functioning somewhat (like with specific activities) but most of time can be functional.
- Anxiety limits functioning regularly but many things are not limited.
- Anxiety limits functioning frequently most of the day, most days.
- Anxiety limits functioning all the time in every activity.
- Comments:

Q20. How could you tell if the person with FXS had less anxiety?

Free response.

Q21. This is the last question for caregivers and professionals. Please provide any additional comments about the person with FXS who seems to be anxious that you would like to add.

Comments.
